# The Welleye: A Conceptual Framework for Understanding and Promoting Wellbeing

**DOI:** 10.3389/fpsyg.2021.716572

**Published:** 2021-10-29

**Authors:** Paul Dolan, Kate Laffan, Laura Kudrna

**Affiliations:** ^1^Department of Psychological and Behavioural Science, London School of Economics and Political Science, London, United Kingdom; ^2^UCD Geary Institute for Public Policy, University College Dublin, Dublin, Ireland; ^3^UCD Economics, University College Dublin, Dublin, Ireland; ^4^Murray Learning Centre, Institute of Applied Health Research, University of Birmingham, Birmingham, United Kingdom

**Keywords:** wellbeing, attention, policy, framework, time use

## Abstract

We present the Welleye – a novel and conceptually clear framework that shows how attention links the objective circumstances of people’s lives and selves to how they spend their time and feel day to day. While existing wellbeing frameworks in policy contain many of the factors included in the Welleye, they all lack attention as the “lens” that determines the impact of these factors on how people feel. Policymakers and organizations can use the Welleye to better understand how people are faring and design and evaluate interventions aimed at making people better off.

## Introduction

There are many ways to determine how well someone’s life is going, and to assess whether and in what ways society is progressing. Economists have long relied on income to proxy for individual wellbeing and gross domestic product as the benchmark for societal progress. Policymakers have additionally focused on objective goods such as health status and social capital. More recently, there have been attempts to capture assessments of how well someone considers their own life to be going overall (evaluations) and how they feel on a day-to-day basis (experiences) using measures of subjective wellbeing (SWB; Graham et al., 2018).

Many frameworks have been designed to help guide how to measure and influence wellbeing. Prominent examples include the Organisation for Economic Cooperation and Development’s (OECD) Better Life Index (Marmot et al., 2012; Durand, 2015), the United Kingdom’s Office for National Statistics’ Wellbeing Dashboard, the Canadian Wellbeing Index of Wellbeing and the Monet System in Switzerland. Many of these frameworks include factors that capture the circumstances of people’s lives, such as their health and education, their evaluations of their lives or aspects of their lives, their time use, and reports of their SWB. One major problem with these frameworks is that they fail to account for a fundamental mechanism through which circumstances, activities and other factors determine our SWB: where our attention is directed (Kahneman et al., 2006).

Attention must be central to any wellbeing framework because the same stimulus can have a very different effect on how people feel depending on the attention allocated to it. For example, how a person’s income affects them will depend on to whom they compare themself to and how they spend their time – and on the attention they pay to these comparisons and experiences. A range of evidence supports the role of attention as a pathway to wellbeing. For example, research on resource scarcity shows that people react differently to stimuli like money and food because they focus more attention on what is least available to them (Shah et al., 2019). According to socio-emotional selectivity theory, older adults have better emotional experiences than younger adults because they focus more attention on positive and less on negative stimuli (Carstensen et al., 2011; Reed et al., 2014). As another example, people adapt differently to changes in their circumstances, such as unemployment or marriage, which is determined in large part by the withdrawal of attention from these changes over time (Diener et al., 2018).

**FIGURE 1:**
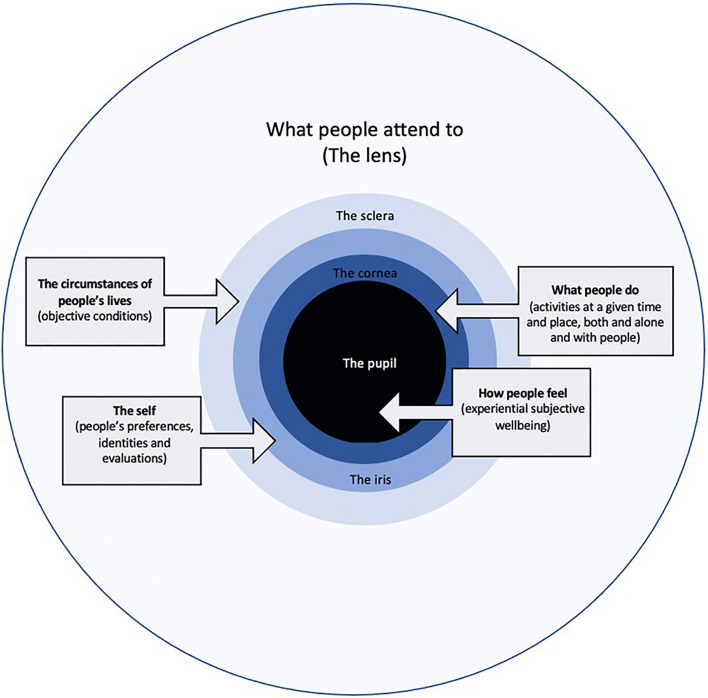
Visualization of the Welleye.

To facilitate conceptual clarity about the relationships between the definitions and determinants of wellbeing, and to illustrate the central role of attention in shaping wellbeing, we propose “The Welleye.” The Welleye is a framework based on the anatomy of an eye that can be used to understand and promote wellbeing in policy and practice. A main contribution of the Welleye is to highlight the attentional “lens” that shapes the factors that are prominent in other wellbeing frameworks, and the role of the lens in determining the impact of these factors on how people feel. We divide the other aspects of wellbeing into three categories. The first category is the objective circumstances of people’s lives, which are located in the sclera of the eye and include age, gender, health and education, among other things. The second category is people’s subjective selves, which includes their preferences, identities, and evaluations, which we locate in the iris. The third category represents people’s activities which are located in time and space and often involve others. The pupil represents experiential SWB (ESWB); namely, feelings day-to-day over the course of their lives. We contend that individuals and societies should ultimately seek to optimize ESWB. See Figure 1 below for a graphical representation of the Welleye. While many existing wellbeing frameworks include some (but not all) of the elements that sit within the sclera, the iris, the cornea, and the pupil, they neglect the role of the lens.

## The Welleye and Existing Frameworks

The Welleye framework uses the anatomy of an eye to illustrate how attention filters the circumstances of people’s lives and their subjective selves to how they spend their time and they feel day to day. In this section, we outline the elements of the Welleye that are represented, to varying degrees, in other wellbeing frameworks: the sclera (objective circumstances), iris (subjective selves), cornea (time-use), and pupil (ESWB).

### The Sclera

The sclera, the supporting wall of the eye, represents the circumstances of people’s lives that serve to enhance or diminish their wellbeing. The sclera consists of a list of objective circumstances that are widely recognized as components of wellbeing (Parfit, 1984). Many fall under the remit of government. These include wider social, political, and economic contexts, including health and social care, transport, education, employment, environmental quality, and housing. Such circumstances are widely included in existing wellbeing frameworks with measures that act as key performance indicators that reflect these overall dimensions. Examples from the OECD’s Better Life Index include household net adjusted disposable income and formal educational attainment. There will be other circumstances featured in the sclera, such as age, height, genetics, biology, the size of social networks, and features of the natural environment. These are captured by frameworks that consider the clinical and social determinants of health and wellbeing (Davies et al., 2014; Røysamb and Nes, 2019).

### The Iris

The iris, the part of the eye unique to each person, reflects individuals’ selves. Economists typically link wellbeing to preferences: people are better off when they can satisfy more of their desires (Harsanyi, 1996; Hausman and McPherson, 2009). The universal inclusion of measures of income and wealth in existing wellbeing frameworks arguably reflects the fact that these measures act as a proxy measures of preference satisfaction given that the greater a person’s economic resources the more of their preferences they can satisfy. As an element of individuals’ selves, preferences belong in the iris. Other individual differences contained within other wellbeing frameworks, such as identities (Akerlof and Kranton, 2000), motivations (Ryan and Deci, 2000), levels of self-esteem and acceptance, eudaimonic reports of functioning, goals, purpose in life (Ryff and Keyes, 1995; Kashdan et al., 2008), personality (Gomez et al., 2009), and attitudes and values, are all situated in the iris. So too are evaluations of life satisfaction, which are closely, but not perfectly, aligned with the preference satisfaction account of wellbeing (Adler, 2012). Life satisfaction, as well as domain specific measures such as health satisfaction, are commonly included in existing frameworks including the UK’s Wellbeing Dashboard (2021) and Switzerland’s Monet (The Monet Indicator System, 2021) system. Many person-environment fit models consider how the iris is situated within the sclera (Caplan, 1987; Lai et al., 2020).

### The Cornea

The cornea captures people’s time-use, as well as the social, situational, and temporal factors associated with daily activities. These factors include who people are with, and when and where an experiences is happening. Elements of time-use are often, but not always, included in existing frameworks. For example, the OECD’s Better Life Index includes a dimension on work-life balance, which is partly assessed using a metric of time spent in personal and leisure care, and another on social connections, which is assessed using a measure of time spent in social interactions. The Canadian Index of Wellbeing (2021) includes other time-use indicators, including for example on commuting time, sleep, and time spent with friends.

### The Pupil

At the center of the eye is the pupil. It represents people’s ESWB, which we define to include hedonic (pleasure-related) feelings (such as joy, pain, or worry) and eudemonic (purpose-related) ones (such as worthwhileness, pointlessness, or futility) (Kahneman and Riis, 2005; National Academies of Science Panel on Measuring Subjective Well-Being in a Policy-Relevant Framework, 2013; Martikainen et al., 2021). This distinction matters (Dolan and Kudrna, 2016). Working, for example, is an activity that is experienced as low in pleasure but relatively higher in purpose (White and Dolan, 2009). How people’s ESWB is measured will, therefore, affect conclusions about how societies can be structured, and individual lives organized, to maximize it. The overarching vision is that people maximize their ESWB over their life course. Each of us will have different inputs that affect how we feel, and each of us will desire and require a different balance of pleasurable and purposeful experiences (Prinz and Bünger, 2012; Dolan, 2014; O’Donnell and Oswald, 2015). Although some frameworks include measures of ESWB, for example reports of happiness and anxiety on the previous day in the UK’s Wellbeing Dashboard, they typically present them alongside factors such as people’s circumstances rather than identifying ESWB as the ultimate goal.

## The Lens: Attention as the Pathway to Experiential Subjective Wellbeing

The idea that people are what they attend to has been around for more than a century (James, 1904), but it has yet to be properly accounted for in wellbeing frameworks for use in policy (Dolan, 2014). As the lens of the eye controls what an individual focuses on, the lens of the Welleye represents what individuals attend to as they live their lives. We contend that attention is the mechanism through which stimuli in the sclera (objective circumstances), iris (subjective selves), and cornea (time-use) ultimately affect how people feel (in the pupil). Building on dual processing models (Dolan, 2014), attention will be allocated consciously and unconsciously, and in various combinations along the spectrum in between, including by attending to attention as in meta-cognitive and meta-awareness approaches (Randall et al., 2014).

### Attention, Circumstances, and Wellbeing

Attention is crucial for understanding the impact of circumstances in the sclera on people’s ESWB. There will be some stimuli that people adapt to because they stop paying attention to them, and others that they do not get used to because they keep paying attention to them. Poverty and unpredictable noise, for example, are both attention-seeking objective conditions that people do not adapt to very easily or quickly because attention is constantly drawn to them over time (Dolan and Kahneman, 2008; Clark et al., 2016). Moreover, people’s circumstances influence the stimuli that are attention-seeking. Research on resource scarcity highlights that when, for example, income or food is scarce, attention tunnels into focus on these resources, with consequences for the choices people make and how they feel (Mullainathan and Shafir, 2013). Attention shapes how circumstances affect how people feel.

### Attention, Subjective Selves, and Wellbeing

Attention is central to the relationships between the elements of the iris and ESWB. Preferences and attitudes will direct what people attend to in their daily lives; for example, people will pay more attention to food’s nutritional content if they are health conscious, and spend more time looking at news stories that align with their political views than those that challenge them (Knobloch-Westerwick et al., 2020). As another example, people with a strong sense of purpose in life are better able to ignore stimuli that get in the way of their goals and concentrate on achieving what feels good to them (Schultz, 2000). Attention also shapes how people view themselves and what they prefer, such as when repeated exposure and attention to advertisements (a circumstance) leads to greater liking of the advertised product (an aspect of subjective selves) (Yagi and Inoue, 2018). Overall, attention explains how aspects of people’s selves affect how people feel.

### Attention, Time-Use, and Wellbeing

The impact of time-use in the cornea on ESWB, including the impacts of what people do, who they are with, and when and where these experiences take place, will all be determined by the attention people pay to those stimuli. We all feel differently when we pay different degrees of attention to the activities, people, and places in our lives. It is easy to see how commuting, for example, can be a very different experience depending on who you interact with and what other activities you engage in while doing so (Epley and Schroeder, 2014). It is possible to engage in more than one activity at any one time, such as when reading a book whilst listening to music on a commuter train, and to feel differently during the same activity at different times, such as the commute on Monday compared to on Friday. Our ESWB is therefore determined not only by the stimuli themselves but by the attention we pay them.

### Summing Up Attention

Attention is the pathway through which circumstances, selves, and activities affect how people feel. Key components of other wellbeing models, such as levels of autonomy, accomplishments, and relationships will affect ESWB differently depending on how much attention is paid to them (Ryan and Deci, 2000; Seligman, 2018). Each element is related to one another in a dynamic system. For example, people in ill-health may engage in less physical activity, potentially further contributing to their ill-health. The strength of the impacts of this reciprocal relationship will depend on how much attention a person pays to their ill-health and their lack of physical activity. Different objective circumstances of life, such as income, will be filtered through attentional lenses with apertures that differ according to aspects of people’s selves, such as their materialism, and how they use their time, such as how often they go shopping (Van Boven, 2005; Rözer et al., 2021). Attention can, therefore, help us categorize and weight the different dimensions of the other elements to understand the determinants of ESWB better.

## Applications

Public policy has largely focused on promoting people’s wellbeing through enhancing the circumstances of people’s lives, and those working within the behavioral sub-set of public policy specifically have sought to promote people’s wellbeing and other societal goals through shaping what people do. While the Welleye clearly recognizes the importance of people’s circumstances, selves, and activities for wellbeing, it identifies that how people feel over their life course is the ultimate outcome that policymakers should be seeking to optimize. Irrespective of debates about the ultimate outcome, attention remains a key ingredient in shaping feelings over the life course.

The Welleye facilitates mapping what matters for people’s wellbeing and understanding how to enable them to improve their ESWB. In what follows, we detail its relevance to two example areas: public policy and organizational practice. The framework is designed to be more broadly applicable, of course, such as to specific policy areas like transport, the environment and housing, and to other contexts, such as therapy, education, and learning. It is beyond the scope of the current work to map out all of the implications of the Welleye framework for policy and practice, and so we highlight three key issues that the Welleye draws out: (1) attention as the mechanism, (2) efforts to directly and indirectly target attention, and (3) adaptation, or the withdrawal of attention.

### Public Policy

The Welleye can assist policymakers to better understand the determinants of wellbeing and the how to promote it. Through the lens of the Welleye, policymakers should be concerned with people’s circumstances, selves, and activities only insofar as they affect how people feel. Importantly, as these relationships are ultimately determined by how these factors shape what people attend to, policymakers should consider this central pathway in deciding what policy should to target and how.

#### Attention as the Mechanism

People’s circumstance, selves, and time-use promote and impede people’s ESWB through the mechanism of attention. Public policy makers working on poverty alleviation, for example, should not only inquire into the effectiveness of a policy at increasing the economic resources available to a target population, but also examine how the policy affects what they attend to, including, for example, intrusive thoughts about money and levels of focus on the trade-offs involved in everyday consumption choices (Gennetian and Shafir, 2015). Identifying attention as the pathway between poverty and ESWB wellbeing helps policymakers to better understand how living in poverty impacts people’s day to day experiences and how to address the negative impacts of poverty on how people feel. Understanding how attention works as a mechanism will therefore improve the effectiveness of anti-poverty initiatives (Moore et al., 2015).

#### Directly and Indirectly Targeting Attention

Policymakers should explore interventions that both indirectly and directly target what people attend to with a view to promoting their wellbeing. Efforts to shape how people spend their time, for example, should target those behaviors that evidence suggests affect people’s attention and promote ESWB. For example, both Attention Restoration Theory and empirical evidence point to the idea that spending time in nature is restorative, i.e., that it reduces mental fatigue and improves concentration, largely because time in nature attracts attention in positive ways (Kaplan, 1995; Ohly et al., 2016). Time in nature has also been shown have positive impacts on people’s ESWB (Nisbet and Zelenski, 2011). There also now exists a range of mental health interventions that aim to promote people’s wellbeing by shaping their attention directly, including those that help mitigate unwanted ruminations and intrusive thoughts (Layard and Clark, 2014) and shape people’s narratives about themselves and their communities (Lloyd-Williams et al., 2018; Tam et al., 2020).

#### Adaptation

Policymakers should aim to better understand the circumstances of people’s lives and the activities they engage in that continue to be attention seeking over time. Factors that deliver a sustained impact on people’s ESWB should be targeted over those that people adapt to more easily. For example, it is more difficult to adapt to mental illness than to having “some problems walking about,” which suggests more resources should be invested in the former than the latter (Dolan and Metcalfe, 2012). This approach raises concerns about the “double jeopardy” argument whereby those who adapt to adversity are given less priority on account of their laudable effort to adapt. This is a vexing moral issue that we cannot aim to resolve here but, suffice to say, that, all else equal, we may wish to prioritize those conditions and circumstances people do not get used to precisely because they are unable to adapt (Menzel et al., 2002).

### Organizational Practice

Organizations can use the attentional lens in the Welleye to better understand the wellbeing of their workforce and how to promote it. This matters not only for employes themselves, but also for other goals, as workers who are happy and well have been shown to perform better (Oswald et al., 2015) and be more creative (Rego et al., 2009), which has organizational and societal benefits.

#### Attention as the Mechanism

The Welleye can inform organizational management by highlighting people’s circumstances, selves, and time-use as inputs into employe wellbeing, and attention as the pathway through which these inputs affect how people feel. For example, in designing a rewards scheme, employers should not just consider how much money to spend on the scheme but also how it will affect what people attend to and how they feel. More income could draw attention to the extrinsic benefits of work and reduce intrinsic motivation to achieve (Ryan and Deci, 2000), and the attention seeking nature of a lump sum bonus once a year may be different to that of more regular but smaller bonuses. Bonuses or rewards that are made public will likely be more attention seeking than those made privately with important consequences for how the recipients feel. Person-fit models work shows that employes’ subjective selves influence the attention they pay to their work circumstances (Caldwell and O’Reilly, 1990; Wrzesniewski and Dutton, 2001), and task variety enhances meaningful work because novelty is attention-seeking (Berg et al., 2013).

#### Directly and Indirectly Targeting Attention

Organizations should also put in place policies and practices that both indirectly and directly target what people attend to with a view to promoting their wellbeing. Consideration of attention could lead to organizational policy changes around employe “attentional energy,” which is a term that refers to the limited capacity of attentional processes such as divided attention, focusing, and recall (Kaplan and Berman, 2010; Dolan, 2014). Attention seeking stimuli such as emails and meetings should be considered, particularly as such stimuli have been negatively linked to ESWB at work (Giurge and Bohns, 2021). In some companies, employes have the “right to disconnect,” which may mean disabling email access after-hours (Henshall, 2021). Organizations could also look to promote wellbeing by encourage their employes to engage in activities that benefit their ability to focus and their wellbeing, such as through sleep hygiene and fatigue management programs (Redeker et al., 2019).

#### Adaptation

Organizations looking to promote employe wellbeing should consider how work environments, tasks, stressors, and other circumstances impact upon what people attend to and how they feel over time. This could vary by employe characteristics such as personality type (Niessen et al., 2010; MacIntyre et al., 2019) and past experience, as well as their age or biology. For example, change management for organizational transitions such as mergers, acquisitions, and restructurings should consider employe’s capacity to adapt (Marks, 2007). Although any changes may initially be attention-seeking, if aspects of the change subsequently remain constant over time, employes are more likely to adjust than if there are further changes (Wilson and Gilbert, 2008).

## Implementation

The Welleye framework can help those trying to promote wellbeing to better understand it and design and evaluate interventions aimed at promoting it. It should be used within a wider implementation framework that considers in detail how interventions are developed, implemented, and evaluated (Damschroder et al., 2009). For example, the Welleye could be a starting point when co-producing a plan of action or reflecting on why there was no effect when one was expected (Osman et al., 2020). Because the Welleye captures complex systems, it can show how wider funding structures and multi-sectoral approaches could be applied in a joined-up way to interactively tackle problems across policy silos. Just as obesity might require changes across many contexts, including meals at school, walks at work, and prices on the shelves in stores (Government Office for Science, 2007), optimizing ESWB requires thinking about these contexts and the relationships between them.

Any optimization must account for the distribution of wellbeing, which could act as a corrective lens to the Welleye. For societal wellbeing, we need to aggregate across individuals and time. This will necessarily involve trading off between equity goals – in which the wellbeing of the worst off is given priority – and efficiency goals – in which the greatest increases in wellbeing possible are emphasized. While the Welleye does not directly speak to the question of what this “social welfare function” should look like, it does help to inform policies to promote wellbeing across the distribution once the relative weights given to each group have been decided.

The operationalization of the Welleye involves measuring each of the elements. There are already well-established measures of many aspects of the Welleye, including naturalistic monitoring tools like experience sampling and the day reconstruction method (Scollon et al., 2003), which capture both activities and momentary feelings in the cornea and the pupil; standard gamble and time trade-off measures, which capture health preferences located in the iris (Fujiwara and Campbell, 2011); and approaches to developing lists of capabilities that capture many of the factors that sit in the sclera (Burchardt and Vizard, 2011). Future research should consider capturing where attention is directed as people go about their daily lives and map this directly to their flow of ESWB over time.

## Conclusion

There are many different wellbeing frameworks in policy, but none of them appropriately consider the role of attention. This limitation means that ways to address misery and promote meaning, fulfilment, and happiness may be missed, such as when objective circumstances are treated as fixed rather than fluid in their impact on ESWB over time. The Welleye illustrates how wider circumstances and subjective selves are filtered through the lens of attention to impact on individuals’ flow of hedonic and eudemonic experiences. Future research should consider mapping how circumstances and selves shift attention in predictable ways. In a substantive sense, the Welleye can serve the vision of a society in which people’s ESWB is optimized across the life course.

## Significance Statement

Existing wellbeing frameworks fail to account for a fundamental mechanism through which people’s circumstances and other stimuli influence how they feel: attention. We propose the “Welleye,” a conceptually clear framework that can be used to understand and promote wellbeing using attention. It links the objective circumstances of people’s lives and their subjective selves to their daily experiences and how they feel day to day. We argue that these factors dynamically interact with one another to promote or impede people’s experiential subjective wellbeing and show how what we attend to is critical to understanding these various relationships. The Welleye framework can be used to design and evaluate interventions aimed at making people better off.

## Data Availability Statement

The original contributions presented in the study are included in the article/supplementary material, further inquiries can be directed to the corresponding author.

## Author Contributions

PD, KL, and LK: conceptualization, resources, and writing original draft and editing. PD: supervision, project administration, and funding acquisition. All authors contributed to the article and approved the submitted version.

## Author Disclaimer

The views expressed are those of the authors and not necessarily those of the United Arab Emirates’ National Program for Happiness and Wellbeing, NIHR, ARC, or the Department of Health and Social Care.

## Conflict of Interest

The authors declare that the research was conducted in the absence of any commercial or financial relationships that could be construed as a potential conflict of interest.

## Publisher’s Note

All claims expressed in this article are solely those of the authors and do not necessarily represent those of their affiliated organizations, or those of the publisher, the editors and the reviewers. Any product that may be evaluated in this article, or claim that may be made by its manufacturer, is not guaranteed or endorsed by the publisher.
