# Corrigendum: The Welleye: A Conceptual Framework for Understanding and Promoting Wellbeing

**DOI:** 10.3389/fpsyg.2022.931869

**Published:** 2022-05-17

**Authors:** Paul Dolan, Kate Laffan, Laura Kudrna

**Affiliations:** ^1^Department of Psychological and Behavioural Science, London School of Economics and Political Science, London, United Kingdom; ^2^UCD Geary Institute for Public Policy, University College Dublin, Dublin, Ireland; ^3^UCD Economics, University College Dublin, Dublin, Ireland; ^4^Murray Learning Centre, Institute of Applied Health Research, University of Birmingham, Birmingham, United Kingdom

**Keywords:** wellbeing, attention, policy, framework, time use

In the original article, there was an error. We failed to express our gratitude to Mohamed Abdullah and Tarek Abu Fakhr who greatly contributed to the initial report which led to the Welleye paper.

A correction has been made to Funding Section.

## Funding

The first incarnation of the Welleye was developed by all three authors as part of a wellbeing policy document for the United Arab Emirates' National Program for Happiness and Wellbeing. The authors would like to express their sincere gratitude to the team who were involved in the policy document, in particular Mohamed Abdullah and Tarek Abu Fakhr. During further development, KL was supported by Marie Skłodowska-Curie Individual Fellowship from the European Commission (Project name: Mind The Gap; Project number: 845342) and LK was supported by the National Institute for Health Research (NIHR) Applied Research Centre (ARC) West Midlands, grant number NIHR200165. The University of Birmingham supported the open access publication of the work.

The authors apologize for this error and state that this does not change the scientific conclusions of the article in any way. The original article has been updated.

## Publisher's Note

All claims expressed in this article are solely those of the authors and do not necessarily represent those of their affiliated organizations, or those of the publisher, the editors and the reviewers. Any product that may be evaluated in this article, or claim that may be made by its manufacturer, is not guaranteed or endorsed by the publisher.

